# Reduction-responsive PEtOz-SS-PCL micelle with tailored size to overcome blood–brain barrier and enhance doxorubicin antiglioma effect

**DOI:** 10.1080/10717544.2017.1402218

**Published:** 2017-11-24

**Authors:** Yuling Li, Li Baiyang, Bu Leran, Wang Zhen, Xie Yandong, Du Baixiang, Zhu Dandan, Zhu Yufu, Liang Jun, Yu Rutong, Liu Hongmei

**Affiliations:** a Insititute of Nervous System Diseases, Xuzhou Medical University, Xuzhou, PR China;; b Brain Hospital, Affiliated Hospital of Xuzhou Medical University, Xuzhou, PR China;; c Jiangsu Key Laboratory of Green Synthetic Chemistry for Functional Materials, School of Chemistry and Chemical Engineering, Jiangsu Normal University, Xuzhou, PR China

**Keywords:** Reduction-responsive, blood–brain barrier, tailored size, anti-glioma effect, glioma

## Abstract

A series of novel reduction-responsive micelles with tailored size were designed and prepared to release doxorubicin (DOX) for treating glioma, which were developed based on amphiphilic block copolymer poly (2-ethyl-2-oxazoline)-b-poly (ε-caprolactone) (PEtOz-SS-PCL) and the micelle size could be regulated by designing the polymer structure. The DOX-loaded PEtOz-SS-PCL micelles had small size and rapid drug release in reductive intracellular environments. Biodistribution and *in vivo* imaging studies in C6 glioma mice tumor model showed that DOX loaded PEtOz-SS-PCL43 micelles with the smallest size had superior accumulation and fast drug release in tumor sites. *In vivo* antitumor activity demonstrated that DOX-loaded PEtOz-SS-PCL43 micelles improved antitumor efficacy in contrast to PEtOz-SS-PCL micelles with larger size toward the orthotopic C6-Luci cells-bearing mice. This study shows great potential in tailoring the micelle size and introducing the responsive bonds or compartment for intracellular drug delivery and release in glioma treatment by designing the architecture of the polymer.

## 1. Introduction

Malignant gliomas are the most common high-grade primary brain tumor with high morbidity, mortality and are poorly responsive to current treatments (Wei et al., 2014). The median survival of patients with glioblastoma seldom exceeds 14.6 months (Minniti et al., 2008). A major obstacle for glioma treatment is to deliver drugs effectively to the tumor cells. The blood–brain barrier (BBB) is a major obstacle to the delivery of drugs into brain tumors (Groothuis, 2000). Thus, most of small molecules with low lipid solubility including brain tumor chemotherapeutics rarely cross the BBB and do not enter into the brain (Pardridge 2007a, b; Biddlestone-Thorpe et al., 2012). Developing some strategies for increasing drug in glioma is a great challenge in glioma therapy.

Over the past years, great efforts have been made to overcome these obstacles. Recently with the development of nanotechnology, nanoparticle therapeutic carriers have provided new opportunities to achieve effective therapeutic distribution at tumor sites (Brunetti et al., 2015; Kunjachan et al., 2015; Stylianopoulos, 2013; Liu et al., 2016). These drug carriers are passively targeted to tumors through the enhanced permeability and retention (EPR) effect, so they are ideally suited for the delivery of chemotherapeutics in cancer treatment. The complexity of glioma, especially the existence of the BBB, higher requirements to the control of nanoparticles size emerged. It has been reported that the vascular endothelial cells and associated pericytes are often abnormal in tumors and some BBB was destroyed in brain tumors (Schneider et al., 2004; Wohlfart et al., 2012). Based on this occasion, the glioma-targeted drug delivery systems are mainly divided into two categories. The drug delivery systems must deliver drugs across the BBB for glioma therapy. When the BBB is intact, the systems that must deliver drugs across the BBB for tumor-targeting are defined as cascade-targeting systems. Other drug delivery systems utilized in high-grade glioma are designated as glioma-targeting systems, which mainly accumulated in the glioma by EPR effect (Mager et al., 2017). Creating more safe and small size drug delivery systems is the key to the success of glioma therapy.

Micelle-based drug delivery system has been proven to be an attractive alternative for the delivery of chemotherapy drugs (Sun et al., 2009; Liang et al., 2014; Chen et al., 2015; Zhang et al., 2016). The particle size of micelles was controlled by adjusting the length and ratio of the hydrophilic and hydrophobic segments. However, to design stimuli-responsive micelles would be more effective for tumor therapy, owing to anticancer drugs released exclusively in tumor tissue or inside tumor cell. The reduction responsibility is widely used stimuli in designing drug delivery system for triggered release (Felber et al., 2012; He et al., 2013). Poly(ethylene glycol) (PEG) currently is the most extensively used polymer in drug delivery as hydrophilic parts. Based on its low dispersity (ĐÐ), biocompatibility and limited recognition by the immune system (stealth behavior), PEG remains the gold standard in polymer-based biomedical applications (Knop et al., 2010). However, formation of PEG-antibodies (Tagami et al., 2010), the accelerated blood clearance of PEG (Koide et al., 2010) and nonbiodegradability of PEG resulting in body accumulation of high-molar-mass PEGs limited the usefulness of PEGs in drug delivery in clinical trials (Pasut & Veronese, 2007). Poly(2-ethyl-2-oxazoline)s, abbreviated as PEtOz, provide higher stability, tunability, and functionalization than PEG, while retaining the requisite features of biocompatibility, stealth behavior and low dispersity (Hsiue et al., 2006; Hoogenboom 2009; Zhao et al., 2015). It is better replacements for PEG in drug delivery. A first PEtOz drug conjugate for treatment of Parkinson’s disease recently entered Phase II clinical trials (Moreadith et al., 2017). Therefore, in this study, PEtOz was chosen as hydrophilic part. Owing to biocompatibility and biodegradability nature of polycaprolactone (PCL), it is extensively studied for controlled drug delivery (Manavitehrani et al., 2016; Senevirathne et al., 2017). Thus, PCL was used as hydrophobic part in our study.

**Scheme 1.**
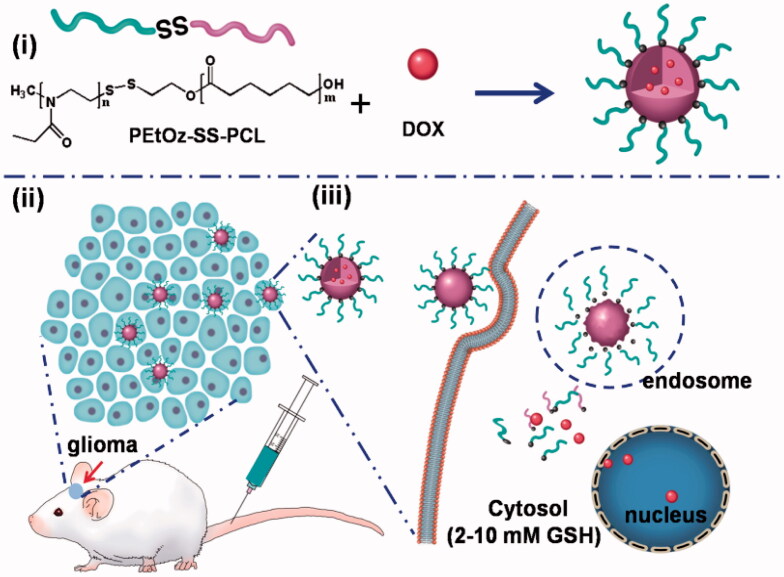
Illustration of reduction-responsive shell-sheddable PEtOz-SS-PCL micelles for triggered DOX delivery *in vivo*. (i) The micelles are assembled from block copolymers PEtOz-SS-PCL; (ii) DOX-loaded micelles efficiently accumulate in C6 glioma tumor; (iii) DOX is quickly released into the cytoplasm triggered by reduction stimuli.

In this study, we synthesized reduction-responsive and size-controllable micelles based on amphiphilic polymer poly (2-ethyl-2-oxazoline) – polycaprolactone (denoted as PEtOz-SS-PCL) to encapsulate DOX for glioma therapy. Control over micelle size is therapeutically important for glioma, as it has been observed that particles with diameter between 20 and 100 nm are effective in penetrating into BBB (Crommelin et al., 2003; Duncan, 2003). In this experiment, PEtOz are hydrophilic parts with fixed polymerization degree and PCL are hydrophobic parts with three different degrees of polymerization (DPs) of 23, 33 and 43. We can obtain micelle with different size by a series of amphiphilic polymer with different DP of PCL block, denoted as PEtOz-SS-PCL23, PEtOz-SS-PCL33 and PEtOz-SS-PCL43. As shown in Scheme 1, the introduction of a disulfide bond between PEtOz and PCL was beneficial to rapidly release DOX in glioma cells. The integration of above features makes PEtOz-SS-PCL an excellent carrier for delivery DOX for glioma therapy.

## 2. Materials and methods

### 2.1. Materials

2-ethyl-2-oxazoline (EtOz), ε-caprolactone (ε-CL) and bis ((2-hydroxyethyl) disulfide) (HES) were dried over CaH_2_ and purified by vacuum distillation. Toluene and tetrahydrofuran (THF) were dried by refluxing over sodium wire, benzophenone as indictor and distilled prior to use. Acetonitrile, N, N-dimethyl formamide (DMF), dichloromethane (DCM), chlorobenzene and methanol were dried by refluxing over CaH_2_ and distilled under dry nitrogen. Methyl-p-toluenesulfonate, potassium thioacetate, 2, 2-dithiodipyridine, dithiothreitol (DTT) and 3-(4, 5-Dimethylthiazol-2-yl)-2, 5-diphenyltetrazolium bromide (MTT) was purchased from Alfa Aeser Co., Ltd. Doxorubicin (DOX) was got from Beijing Zhongshuo Pharmaceutical Technology Development Co., Ltd. All other chemicals were of analytical grade and used without further purification.

### 2.2. Characterization

The ^1^H NMR spectra were recorded on a Unity Inova 400 spectrometer operating at 400 MHz using chloroform-*d*. The molecular weight and polydispersity of copolymers were determined by a PL GPC 50 instrument equipped with Jordi GPC columns (10E4, 2 M) following a differential refractive-index detector (PL-RI). The measurements were performed using DMF as the eluent at 50 °C and a sense of narrow polystyrene standards for the calibration of the columns. The size and zeta-potential of micelles was determined by a Zetasizer Nano-ZS from Malvern Instruments. Transmission electron microscopy (TEM) measurement was performed on a FEI Tecai GT 12 operated with an accelerating voltage of 200 KV. The absorbance of each well in MTT assays was measured using a Synergy H4 Hybrid Multi-Mode Microplate Reader. The fluorescence measurement of doxorubicin was performed using F-4600FL spectrophotometer at 298 K. Flow cytometric analysis using a BD FACSCalibur flow cytometer.

### 2.3. Synthesis of PEtOz-SS-PCL

The copolymers of poly(2-ethyl-2-oxazoline) pyridyl disulfide (PEtOz-SS-Py) were prepared following a procedure reported by Ging-Ho Hsiue (Hsiue et al., 2006) and PCL-SH were synthesized by ring-opened polymerization of ε-CL using HES as initiator, then reacted with DTT, following a procedure reported previously (Sun et al., 2009). The synthesis of amphiphilic polymer PEtOz-SS-PCL43 was used as an example in the following procedure. The PCL-SH (150 mg, 0.0302 mmol) was added into a DCM (6 mL) solution of PEtOz-SS-Py (195.93 mg, 0.0362 mmol) under a nitrogen atmosphere at room temperature and adjusted the pH to 2.5 by adding acetic acid. The reaction was allowed to proceed under stirring for 48 h. The product PEtOz-SS-PCL polymer was isolated into cold diethyl ether, filtration and washing with cold methanol several times to remove excess PEtOz-SS-Py and vacuum-dried to yield the product. The yield of PEtOz-SS-PCL was about 30%.

### 2.4. Preparation of PEtOz-SS-PCL micelles and physicochemical characterization

Micelles of PEtOz-SS-PCL were prepared under stirring by dropwise 1.2 mL double distilled water to 0.5 mL THF solution of block copolymer (0.4 wt%) at room temperature, and removed THF thoroughly by extensive dialysis against PB (10 mM, pH 7.4) for 24 h.

Transmission electron microscopy (TEM) was used to detect particle morphology. Before micelles were measured, 10 μL fresh micelles sample was placed onto carbon-coated copper grids, and micelles were air-dried and then negatively stained by a 2% sodium phosphotungstate solution.

Dynamic light scattering (DLS) measurements were performed in aqueous solution using a Malvern Zetasizer Nano ZS apparatus. To evaluate particle size, the intensity-weighted Z-average of the particle diameter is reported in nm.

The critical micelle concentration (CMC) of polymer PEtOz-SS-PCL was determined by fluorescence spectrometer (FL 4600) using pyrene as a fluorescence probe. The concentration of PEtOz-SS-PCL micelle was changed in a range from 1.0 × 10^−5^ to 0.1 mg/mL, and the pyrene concentration was 0.6 μM. The excitation wavelength of fluorescence spectra was fixed at 330 nm, and then, the emission fluorescence at 372 nm and 383 nm was recorded, and the CMC value was obtained as the interaction point by extrapolating the intensity ratio I_372_/I_383_ at above test concentrations.

### 2.5. *In vitro* cellular uptake

To evaluate the cellular uptake of DOX-loaded PEtOz-SS-PCL micelles, flow cytometric analysis was used. C6 cells at a density of 5 × 10^4^ were cultured in 12-well tissue culture plates and cultured at 37 °C in a 5% CO_2_ humidified atmosphere with DMEM containing 10 vol % FBS for 24 h. Later, old medium was replaced with fresh medium. Then, the cells were treated with DOX loaded PEtOz-SS-PCL23, PEtOz-SS-PCL33, PEtOz-SS-PCL43 nanoparticles and PBS at DOX concentration of 1 μgmL^−1^ for 2 h. The cells were trypsinized, washed three times with cold PBS and resuspended in 500 μL cold PBS for flow cytometric analysis using a BD FACSCalibur flow cytometer.

### 2.6. *In vitro* drug release

The *in vitro* release of DOX from PEtOz-SS-PCL micelles was using a dialysis tube (MWCO 12000) incubated at 37 °C for 24 h in two different media, that is, PB (10 mM, pH 7.4) with 10 mM DTT and PB (pH 7.4, 10 mM). In order to acquire sink conditions, drug release studies were performed at low drug-loading contents (ca. 0.5 wt%), 0.7 mL DOX-loaded micelle solution dialysis against 20 mL of the same medium. At desired time intervals, 6 mL release media were taken out and replenished with an equal volume of fresh media. The concentration of DOX was determined by fluorescence (FL4600) measurements (excitation at 480 nm). To determine the amount of DOX released, calibration curves were run with DOX/PB buffer solutions with different DOX concentrations at pH 7.4. The emission at 600 nm was recorded. The release experiments were performed in triplicate. The results are presented as the average ± SD.

### 2.7. Reduction-responsive size change of PEtOz-SS-PCL micelles

Reduction induced size change of PEtOz-SS-PCL micelles was measured by DLS, which was performed in response to PB buffer (pH 7.4) with or without reductive agent (10 mM DTT) at 37 °C. Typically, PEtOz-SS-PCL43 micelles (1.5 mL) in PB buffer (pH 7.4, 10 mM) were first removed oxygen by bubbling with nitrogen, then, DTT was added to obtain a final concentration of 10 mM. The above mixture was placed in a shaking bed with a speed of 200 rpm at 37 °C. The size was measured by DLS at predetermined time points.

### 2.8. Cell viability assays

The cytotoxicity of PEtOz-ss-PCL23, PEtOz-ss-PCL33, PEtOz-ss-PCL43 and DOX was detected by the 3-(4, 5-dimethylthiazol-2-yl)-2,5-diphenyltetrazolium bromide (MTT) assay. Three thousand C6 cells were seeded in 96-well tissue culture plate and cultured at 37 °C in a 5% CO_2_-humidified atmosphere in 150 μL DMEM containing 10 vol% FBS for 24 h. Then, free DOX and DOX-loaded PEtOz-SS-PCL micelles at different DOX concentrations were added and incubated for another 48 h. After then 10 μL MTT reagent (5 mg mL^−1^) was added to the each well and incubated for an additional 4 h. Medium was removed lightly and 150 μL DMSO was added to each well for 15 min to dissolve purple formazan. At last quantification, measurements (optical density) were obtained at a wavelength of 480 nm using a spectrophotometric analysis.

### 2.9. Mice

ICR mice (male, 18–20 g) were purchased from Beijing HFK Bioscience Co., Ltd. (Beijing, China). All animals received care in compliance with the guidelines in the Guide for the Care and Use of Laboratory Animals. All procedures were approved by Xuzhou Medical University of China Animal Care and Used committee.

Glioma-bearing ICR mice were prepared by intracranial injection (striatum, 1.8 mm right lateral to the bregma and 3 mm of depth) of 1 × 10^5^ C6-Luci cells suspended in 4 µL of L15 medium into male ICR mice (Li et al., 2014; An et al., 2015). After 7 days of xenograft glioma, nude mice were randomly divided into five groups (*n* = 10) and housed in a controlled temperature room with regular alternating cycles of light and darkness.

### 2.10. *In vivo* distribution of DOX-loaded PEtOz-SS-PCL

Glioma-bearing ICR mice were first injected with freshly prepared luciferin substrate and imaged with the Xenogen IVIS Spectrum optical imaging device to prove to have similar volume tumors in the brain after 7 days of xenograft glioma. After that, glioma-bearing ICR mice were injected intravenously with free DOX, DOX loaded PEtOz-SS-PCL23, PEtOz-SS-PCL33 and PEtOz-SS-PCL43 micelles through the tail vein at the dose of 3 mgkg^−1^ DOX per animal. PBS was injected as control. Then, at 4 h after administration, the mice were sacrificed, and the glioma model brains as well as other principal organs (heart, liver, spleen, lung and kidney) were excised carefully and visualized under the *in vivo* real-time fluorescence imaging system.

The excised glioma model brains were then fixed with 4% paraformaldehyde for 72 h and further dehydrated in sucrose solution. Slices of 20 *µ*m thickness were prepared and stained with DAPI for 10 min at room temperature. The slices were observed under fluorescence microscopy and photographed (Olympus, Japan).

### 2.11. *In vivo* antiglioma efficacy

To verify the treatment efficacy of the free DOX, DOX-loaded PEtOz-SS-PCL23, PEtOz-SS-PCL33 and PEtOz-SS-PCL43, 40 C6 tumor-implanted mice were randomized into five groups. The mice were intravenously injected by free DOX, DOX-loaded PEtOz-SS-PCL23, PEtOz-SS-PCL33 and PEtOz-SS-PCL43 at DOX dose of 3 mgkg^−1^ every other day for 8 days. The tumor size was monitored by Xenogen IVIS Spectrum optical imaging device (Caliper Life Sciences) at day 10, 17, 24. The survival time and body weight of mice were measured. After the last treatment, the mice were euthanized and the hearts were collected. The hearts were washed with saline and fixed in the 4% paraformaldehyde. The sections of hearts were stained with hematoxylin and eosin and observed under fluorescence microscopy and photographed (Olympus, Japan).

### 2.12. Statistical analysis

Statistical analysis was performed using the Student’s *t*-test with *p* < .05 as significant difference. The experimental results were given in the format of mean, mean ± SD in the figures.

## 3. Results and discussion

### 3.1. Synthesis of PEtOz-SS-PCL

**Figure 1.**
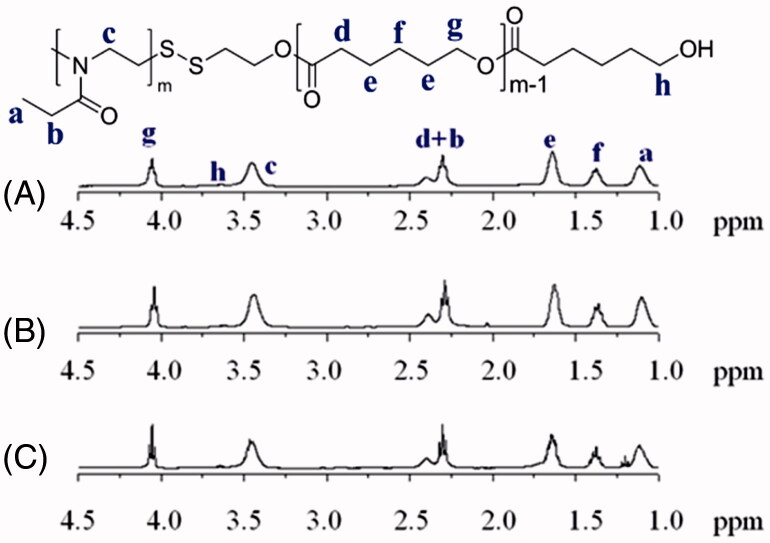
^1^HNMR spectra (400 MHz, C*D*Cl_3_) of PEtOz-SS-PCL23 (A), PEtOz-SS-PCL33 (B) and PEtOz-SS-PCL43 (C).

A series of novel amphiphilic PEtOz-SS-PCL block copolymer was synthesized through exchange reaction between poly (2-ethyl-2-oxazoline) pyridyl disulfide (PEtOz-SS-Py) and mercap to PCL (PCL-SH) in dichloromethane with molar ratio of 1.2/1 (Figure S1, Table 1). Figure 1 showed both hydrophilic comparts of PEtOz (δ 1.10, δ 3.45) and hydrophobic comparts of PCL (δ 1.37, δ 1.65, δ 2.30, δ 4.05) in ^1^H NMR spectra. The integral ratio of signals at δ 1.10 and δ 4.05 manifested an equivalent coupling of PEtOz and PCL. The DP of PEtOz and PCL was calculated from the ^1^H NMR end group analysis (Figure S2) (Zhang et al., 2010; Zhu et al., 2011; Yang et al., 2012). Moreover, GPC results showed that the resultant copolymers PEtOz-SS-PCL had a narrow distribution of molecular weight after the exchange reaction between PEtOz-SS-Py and PCL-SH, with a PDI of 1.27, 1.17, 1.21, respectively (Figure S3). Therefore, these results showed we have synthesized diblock copolymer PEtOz-SS-PCL successfully. These block copolymers were prepared by varying the length of PCL block, while the hydrophilic PEtOz block was fixed. The molar ratio of the initial monomer and initiator concentration were 43: 1, 33: 1, 23: 1 respectively, resulting in different lengths of PCL block. The molecular weight and a PDI of block copolymers PEtOz-SS-PCL were determined by GPC (Figure S4) and summarized in Table 1.

**Figure 2.**
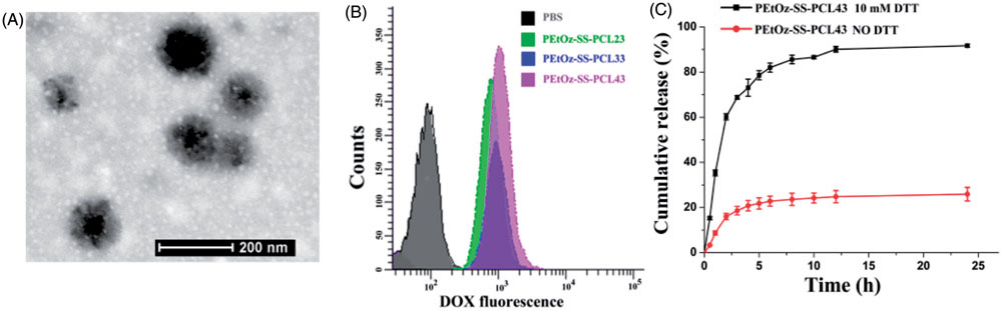
Characteristics of PEtOz-SS-PCL micelles. (A) TEM reveals the morphological structure of PEtOz-SS-PCL43 micelles. (B) Cellular uptake of DOX-loaded PEtOz-SS-PCL23, PEtOz-SS-PCL33 and PEtOz-SS-PCL43 micelles. (C) Reduction-triggered drug release from DOX-loaded PEtOz-SS-PCL43 micelles.

**Table 1.** Molecular characteristics of PEtOz-SS-PCL.

Block copolymers	*M*_n_ (design)	*M*_n_^a^ (^1^HNMR)	*M*_n_^b^ (GPC)	PDI^b^ (GPC)
PEtOz-SS-PCL43	5400–5100	5200–4900	4200–6400	1.27
PEtOz-SS-PCL33	5400–3700	5200–3600	4200–4300	1.17
PEtOz-SS-PCL23	5400–2600	5200–2400	4200–2500	1.21

^a^ determined by ^1^H NMR.

^b^ determined by GPC.

*M_n_* and *M*_W_/*M*_n_ was determined by GPC measurements in DMF (1.0 mL min^−1^, 30 °C, and polystyrene standards).

### 3.2. Characterization of PEtOz-SS-PCL micelles

TEM examination revealed that the PEtOz-SS-PCL43 micelles were dispersed as individual particles with a spherical shape (Figure 2(A)). DLS analysis showed that the average particle size of PEtOz-SS-PCL decreased with the increase of the polymerization degree of PCL, which might due to increasing the concentration of hydrophobic chain. From Table S1, it can be seen that the sizes of the PEtOz-SS-PCL23, PEtOz-SS-PCL33 and PEtOz-SS-PCL43 are 160.2 ± 1.4 nm, 140.6 ± 1.6 nm and 97.3 ± 1.8 nm, respectively. While the size of the DOX-loaded PEtOz-SS-PCL23 and PEtOz-SS-PCL33 are 162.4 ± 0.1 and 137.8 ± 1.3, respectively. Thus, the loading of DOX in PEtOz-SS-PCL23 and PEtOz-SS-PCL33 do not increase the micelles size significantly. However, the size of DOX-loaded PEtOz-SS-PCL43 are smaller than these of corresponding blank micelles, it is 88.4 ± 2.7 nm. The strong attractive hydrophobic interactions between the encapsulated drug and inner core causes the reduced micelle size (Ding et al., 2013; Shi et al., 2015). The physical properties and loading capacity of PEtOz-SS-PCL23, PEtOz-SS-PCL33 and PEtOz-SS-PCL43 were listed in Table S1. The cytotoxicity of PEtOz-ss-PCL23, PEtOz-ss-PCL33 and PEtOz-ss-PCL43 was evaluated with an MTT assay against C6 cells. As shown in Figure S5, surviving cell viability was greater than 90% with 1 mgmL^−1^, demonstrating that the cytotoxicity of these micelles is fairly low. These results suggested that PCL with DP of 43 had the smallest size. So, we used the polymer PEtOz-SS-PCL43 for the following drug release experiment.

CMC is an important property for micelle. We use pyrene as hydrophobic fluorescence probe to measure the CMC value (Figure S6). The CMCs of PEtOz-SS-PCL copolymers were in the range of 5.18–8.29 mgL^−1^, which was lower than those of micelles formed by amphiphilic polymer with similar molecular weight, such as PEG-PCL and PEG-SS-PCL (Table S1) (Sun et al., 2009). The lower CMC values also suggested the micelles self–assembled from PEtOz-SS-PCL were stable upon dilution under physiological conditions, which is beneficial for the drug delivery application *in vivo*. The CMCs of PEtOz-SS-PCL micelles decreased with an increasing amount of hydrophobic PCL block, because the micelle with longer hydrophobic PCL block which played an important role in the stability of the micelle were prone to form. We also studied the effects of size on the cellular uptake efficiency of PEtOz-SS-PCL23, PEtOz-SS-PCL33 and PEtOz-SS-PCL43 micelles by flow cytometry. As shown in Figure 2(B), PEtOz-SS-PCL43 with size around 80 nm were taken up by tumor cells far more than larger micelle of PEtOz-SS-PCL23 and PEtOz-SS-PCL33. The result indicated that the cellular uptake process of PEtOz-SS-PCL micelles highly depended on the size of micelles and small size micelles exhibited high uptake efficiency.

The *in vitro* release of DOX from DOX-loaded PEtOz-SS-PCL43 micelles was studied at 37 °C in PB buffer (pH 7.4) with or without 10 mM DTT. The disulfide bond located between the hydrophilic compartment and hydrophobic compartment would break in the reductive conditions (10 mM DTT) which is mimicking the intracellular cytopolasm (Figure 2(C)). The reduction-responsive drug loaded PEtOz-SS-PCL43 micelle has great potential using as a drug delivery system for the treatment of tumor.

We also used different buffer and reducing reagent DTT to investigate the reduction sensitivity of PEtOz-SS-PCL micelle. The size change of PEtOz-SS-PCL43 micelle was tracked by DLS (Figure S7). Here, we used PB buffer (10 mM, pH 7.4) to simulate the intracellular condition, respectively. The DLS results showed that the micelle size fastly increased from about 100 nm to hundreds of nanometers after 4.0 h in the presence of 10 mM DTT, indicating that the PEtOz-SS-PCL micelle is sensitive to reductive condition. This phenomenon attributed to the reason that reduction sensitivity caused by the disulfide bond between hydrophilic and hydrophobic parts of polymer PEtOz-SS-PCL, which resulted in the disassembly of micelle.

### 3.3. Cell viability assays

**Figure 3.**
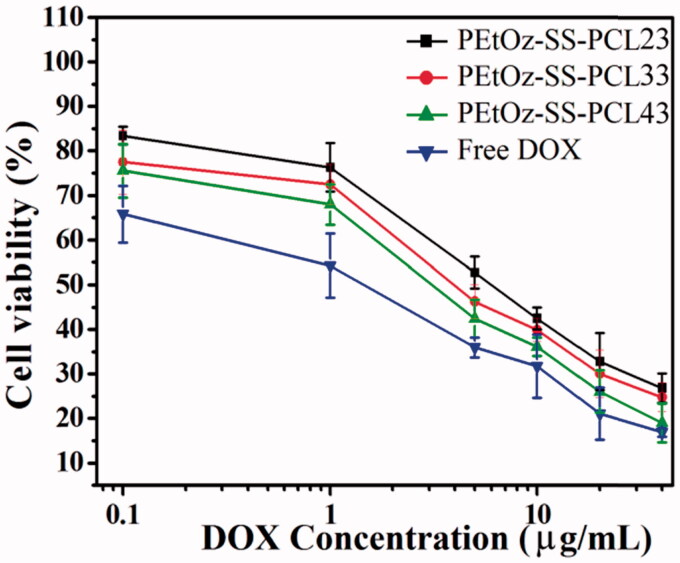
Antiglioma activity of DOX-loaded PEtOz-SS-PCL micelles and free DOX as a function of DOX dosages. The C6 cells were incubated with DOX-loaded micelles or free DOX for 48 h. Means ± SD (*n* = 6).

The *in vitro* cytotoxicity of DOX-loaded PEtOz-SS-PCL micelles against C6 cells was evaluated by MTT assay. DOX-loaded PEtOz-SS-PCL43 micelles presented the significantly enhanced cytotoxicity compared with DOX-loaded PEtOz-SS-PCL23 and DOX-loaded PEtOz-SS-PCL33 at all the DOX concentrations studied (Figure 3). IC_50_ of DOX-loaded PEtOz-SS-PCL43 micelles was 6.67 μg mL^−1^ on C6 cells, which was lower than that of DOX-loaded PEtOz-SS-PCL23 and DOX-loaded PEtOz-SS-PCL33 with IC_50_ value of 45.68 and 16.16 μg mL^−1^, respectively. It was suggested that higher uptake efficiency of DOX-loaded PEtOz-SS-PCL43 compared with DOX-loaded PEtOz-SS-PCL23 and DOX-loaded PEtOz-SS-PCL33 provided higher cytotoxic activity toward the glioma cells. The difference of uptake efficiency for drug-loaded micelle should assign to the different size of drug-loaded micelle, which was also in line with the cellular uptake result of flow cytometry. This phenomenon also demonstrated that the micelle size is one of the key point for cell endocytosis, the smaller size is beneficial for the cell uptake in this procession, and further released cargo under reductive condition in plasma, resulting in different *in vitro* cytotoxicity. Obviously, *in vitro* cytotoxicity of DOX-loaded PEtOz-SS-PCL micelles can be adjusted by tailoring the micelle size.

### 3.4. *In vivo* glioma distribution of PEtOz-SS-PCL

**Figure 4.**
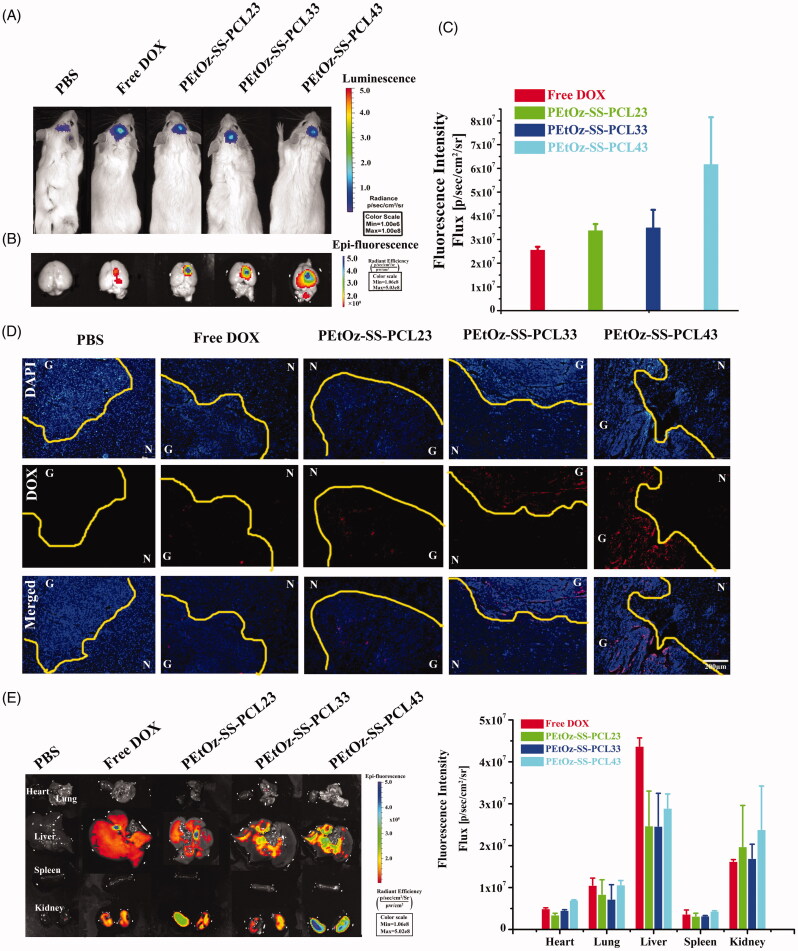
DOX distribution in the glioma after intravenous injection to mice with either free DOX or DOX-loaded PEtOz-SS-PCL micelles. (A) Bioluminescence of luciferase expressing tumor cells 10 min after ip injection of luciferin solution. (B) Fluorescence image of free DOX and DOX-loaded PEtOz-SS-PCL micelles in an removal of the mice brains. (C) The quantitative analysis of DOX in excised mice brains. (D) Fluorescent microscope images show the distribution of DOX in glioma after intravenous injection of free DOX and DOX-loaded PEtOz-SS-PCL micelles. Scale bar: 200 mm. G: Glioma; N: Normal brain tissues. The dashed line = boundary of the glioma. (E) Fluorescent image of tissues distribution of free DOX and DOX-loaded PEtOz-SS-PCL micelles (at DOX concentration 3 mg kg^−1^) were intravenously injected to C6-Luci bearing ICR mice and quantitative analysis of DOX in tissue.

The *in vivo* targeting of glioma and localization in various organs of DOX-loaded PEtOz-SS-PCL were observed in orthotopic C6-Luci cells-bearing mice by the IVIS kinetic imaging system (Caliper Life Sciences, Hopkinton, MA). First, the brain tumor model was developed in ICR mice using stereotactic intracranial injection of ∼1 × 10^5^ C6-Luci cells into the primary somatosensory cortex. After 7 days post implantation, the bioluminescence signals were analyzed by *in vivo* bioluminescence imaging. As shown in Figure 4(A), the *in vivo* image finding was confirmed the existence of brain glioma. And then, DOX-loaded PEtOz-SS-PCL and free DOX were administered intravenously to orthotopic implantation model of glioma cells in ICR mice, and was examined after 4 h by an *in vivo* imaging system and fluorescence microscopy, respectively. The fluorescence intensity of free DOX in the brain was negligible, indicating that free DOX barely crossed the BBB in the mice. DOX loaded PEtOz-SS-PCL micelles with different PD of PCL had stronger DOX fluorescence than free DOX (Figure 4(B,C)). Compared to DOX-loaded PEtOz-SS-PCL23, PEtOz-SS-PCL33 treated mice, the strongest DOX fluorescence was found at the mice’s tumor region treated with DOX-loaded PEtOz-SS-PCL43. These results indicated that PEtOz-SS-PCL43, which has the smallest nanosize could effectively enter into glioma by EPR effect. The tumor distribution of PEtOz-SS-PCL and free DOX was also confirmed by frozen tumor tissue section observed by fluorescence microscope (Figure 4(D)). Brain tumor tissue was identified by areas of hypercellularity as evident from DAPI-stained cell nuclei shown in blue (Figure 4(C)). The C6-Luci bearing mice treated with PEtOz-SS-PCL43 had more DOX fluorescence in brain tumor than treated with other groups, indicating that PEtOz-SS-PCL43 could effectively deliver DOX to brain tumor. After that, C6-Luci bearing brains and major organs were excised for *ex vivo* imaging to reveal the tissue distribution. A clearer result was found in the ex-brain imaging (Figure 4(E)). Besides, the amount of PEtOz-SS-PCL was less in liver compared to free DOX group, suggesting a lower toxicity to liver. While the DOX fluorescence in heart, spleen, lung and kidney was similar in DOX-loaded PEtOz-SS-PCL23, PEtOz-SS-PCL33 and PEtOz-SS-PCL43 groups. These findings demonstrated that PEtOz-SS-PCL43 group with the smallest nanosize could effectively transport the DOX across the BBB and could result in the highest cellular uptake of DOX by glioma.

**Figure 5.**
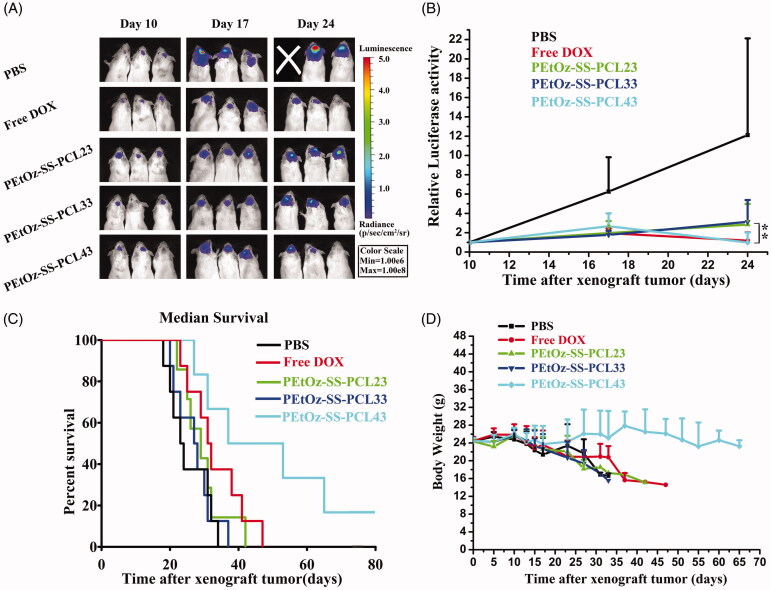
*In vivo* efficacy in C6-Luci glioma model in mice. C6-Luci-bearing mice received three injections of PBS, Free DOX, DOX-loaded PEtOz-SS-PCL23, DOX-loaded PEtOz-SS-PCL33 and DOX-loaded PEtOz-SS-PCL43 at a dose of 3 mgkg^−1^ DOX. (A) Bioluminescent signal change correlating to tumor growth over time following inoculation. (B) Quantification of the tumor bioluminescence signal (*n* = 5 mice per group). (C) Kaplan–Meier survival curve of the mice. Data are presented as Mean ± SD (*n* = 8, **p<.01). (D) Body weight change.

We next evaluated the therapeutic efficacy of locally administered free DOX, DOX-loaded PEtO_Z_-SS-PCL23, DOX-loaded PEtO_Z_-SS-PCL33 and DOX-loaded PEtO_Z_-SS-PCL43 (the concentration of DOX 3 mg kg^−1^) by intravenous injection at 11 days established in ICR mice by stereotactic intracranial injection of ∼1 × 10^5^ C6-GFP-Luci human glioma cells into the cortex (Figure 5(A)). Tumor growth was measured *in vivo* using bioluminescence imaging. Tumors in animals in the control group (PBS) grew rapidly (Figure 5(B)). By day 24, the tumor loaded in PBS group, as reflected by bioluminescence measurements, was 12.11-fold higher than at day 10. The tumor growth rate in mice that treated with free DOX, DOX-loaded PEtO_Z_-SS-PCL23, DOX-loaded PEtO_Z_-SS-PCL33 and DOX-loaded PEtO_Z_-SS-PCL43 were 1.18-fold, 2.85-fold, 3.13-fold and 0.99-fold higher than at day 10, respectively. From this result, we found that DOX-loaded PEtOz-SS-PLC43 had the highest antiglioma activity, due to DOX-loaded PEtO_Z_-SS-PLC43 delivered more DOX to glioma than DOX-loaded PEtO_Z_-SS-PLC23 and DOX-loaded PEtO_Z_-SS-PLC33, which was confirmed by the distribution of DOX fluorescence in glioma.

To further estimate the antitumor efficacy, the body weight and overall survival of the glioma-bearing mice were assessed (Figure 5(C,D)). As shown in Figure 6(C), treatments with DOX-loaded PEtOz-SS-PCL23 and DOX-loaded PEtO_Z_-SS-PCL33 did little in improving mice survival, registering a median survival of 29 days and 27.5 days versus 23.5 days for the PBS-treated group. Although remarkable tumor inhibition was observed in DOX-treated group, no benefit of median survival time emerged. Compared to the DOX-treated group (median survival, 31.5 days), DOX-loaded PEtOz-SS-PCL43-treated groups possessed prolonged survival times. The median survival time was 45 days for DOX-loaded PEtOz-SS-PCL43 treated group. The dominance of DOX-loaded PEtOz-SS-PCL43 was also reflected on the body weight change. The body weight showed a slow decrease of DOX-loaded PEtOz-SS-PCL43, while other groups had a rapid decrease (Figure 5(D)). All the above results demonstrated DOX-loaded PEtO_Z_-SS-PCL43 had a predominant therapeutic efficacy for glioma, due to its the smallest nanosize.

### 3.5. H and E staining analysis

DOX is a highly effective and widely used chemotherapeutic drug to cure various types of cancer; however, its effectiveness is limited by its cardiac toxicity (Cho et al., 2012; Guan et al., 2013; Subburaman et al., 2014). In order to further evaluate the cardiotoxicity of DOX and DOX-loaded PEtO_Z_-SS-PCL treatment, the histological analysis of the cardiac tissues was tested. As shown in Figure S8, histological examinations did not show any myocardial lesions in the group of mice treated with free DOX, DOX-loaded PEtOz-SS-PCL23, DOX-loaded PEtOz-SS-PCL33 and DOX-loaded PEtOz-SS-PCL43, suggesting that the treatment with free DOX, DOX-loaded PEtOz-SS-PCL23, DOX-loaded PEtOz-SS-PCL33 and DOX-loaded PEtOz-SS-PCL43 at the experimental dosage did not damage the heart of the mice during the experimental period.

## 4. Conclusions

In summary, we have successfully synthesized reduction-responsive and controlling size DOX-loaded PEtOz-SS-PCL micelles to encapsulate DOX for glioma therapy. The DOX-loaded PEtOz-SS-PCL43 micelles showed the smallest size distribution and effectively entered into glioma *in vivo*. Moreover, treatment with DOX-loaded PEtOz-SS-PCL43 micelles inhibited significantly brain tumor growth in ICR mice orthotopic glioma model compared to other groups. Therefore, PEtOz-SS-PCL43 micelles can be potentially applied as a safe and efficient drug delivery for glioma treatment.

## Supplementary Material

IDRD_Mei_et_al_Supplemmental_Contenent.docx
