# Supplementary material for: Reduction-responsive PEtOz-SS-PCL micelle with tailored size to overcome blood–brain barrier and enhance doxorubicin antiglioma effect
Source: Drug Deliv. 2017 Nov 24;24(1):1782–90. doi: 10.1080/10717544.2017.1402218 (PMC8241033; doi:10.1080/10717544.2017.1402218)
Supplement: IDRD_Mei_et_al_Supplemmental_Contenent.docx [file IDRD_A_1402218_SM4389.docx]

(Supporting Information)

Reduction Responsive PEtOz-SS-PCL Micelle with Tailored Size to Overcome Blood Brain Barrier and Enhance Doxorubicin Anti-glioma Effect

Yuling Li^b#^, Baiyang Li^a#^, Leran Bu^b#^, Zhen Wang^a^, Yandong Xie^a^, Baixiang Du^b^, Dandan Zhu^b^ , Yufu Zhu^a^, Jun Liang^a*^, Rutong Yu^a*^ and Hongmei Liu^a*^

^a^ Insititute of Nervous System Diseases, Xuzhou Medical Universit, Xuzhou, Jiangsu, PR China; Brain Hospital, Affiliated Hospital of Xuzhou Medical University, Xuzhou, PR China;

^b^Jiangsu Key Laboratory of Green Synthetic Chemistry for Functional Materials, School of Chemistry and Chemical Engineering, Jiangsu Normal University, Xuzhou, PR China;

E-mail: liuhongmei816@sina.com

^#^These authors contributed equally to this work.

**Synthesis of poly(2-ethyl-2-oxazoline)pyridyl disulfide (PEtOz-SS-Py)**

The copolymers of poly(2-ethyl-2-oxazoline) pyridyl disulfide (PEtOz-SS-Py) were prepared following a procedure reported by Ging-Ho Hsiue (Hsiue et al., 2006). ^1^2-Ethyl-2-oxazoline (10 g, 100.9 mmol) and a solution of methyl *p*-toluenesulfonate (0.340 g, 1.834 mmol) were added into dry acetonitrile. The mixture was heated to 100 ^o^C and stirred for 24 h under dry nitrogen. The living PEtOz polymer chains were terminated by adding potassium thioacetate (0.357 g, 3.08 mmol) into the solution at 0 ^o^C and then stirred for 24 h again. The polymer solution was filtered through the silica gel and was isolated by precipitation into diethyl ether. The copolymers poly(2-ethyl-2-oxazoline) thioacetate (PEtOz-SAc) were purified by dialysis against distilled water for two days by using dialysis membrane (MWCO: 3500 g mol^-1^) and isolated by freeze-drying. The product PEtOz-SAc was characterized by ^1^H NMR (C*D*Cl_3_, as solvent). PEtOz-Sac (1.0 g, 0.2 mmol) and 2, 2^’^-dithiodipyridine (222 mg, 1 mmol) were dissolved in ammonia/methanol solution. The mixture solution was stirred at room temperature in a dry nitrogen atmosphere for 24 h. After removing the solvent, the solid residue was dissolved in dichloromethane and isolated by precipitation into diethyl ether, then vacuum-dried to yield a yellow product. Yield: 79%. The product was characterized by ^1^H NMR (C*D*Cl_3,_ as solvent).

**Synthesis of PCL-SH**

PCL-SH were synthesized by ring-opened polymerization of *ε*-CL using HES as initiator, then reacted with DTT, following a procedure reported exactly **(**Sun et al., 2009**)**. *ε*-CL (7.22 g, 63.27 mmol) and HES (101.5 mg, 0.659 mmol) were added into dry toluene (42 mL) under a nitrogen atmosphere and stirred the solution by an electromagnetic stirrer. After completely dissolved, stannous octoate (Sn (Oct.)_2_, 80.9 mg, 0.199 mmol) was added into the solution. The mixture was heated to 100 ^o^C using an oil bath and kept stirring for 24 h. The reaction was terminated by adding excess HCl. The PCL-SS-PCL polymer was isolated in cold diethyl ether, filtration and vacuum-drying for 24 h. Yield: 80 %.

DTT (93.48 mg, 0.606 mmol) was added into DMF (7 mL) solution of PCL-SS-PCL (0.80 g, 0.08 mmol) under stirring. The reaction was allowed to proceed for 24 h at 25^o^C. The resulting polymer PCL-SH was precipitated in cold diethyl ether, filtrated under nitrogen flow and dried in vacuum oven. Yield: 72 %. The product was characterized by ^1^H NMR (C*D*Cl_3,_ as solvent).

**Figure S1.** Synthesis of PEtOz-SS-PCL


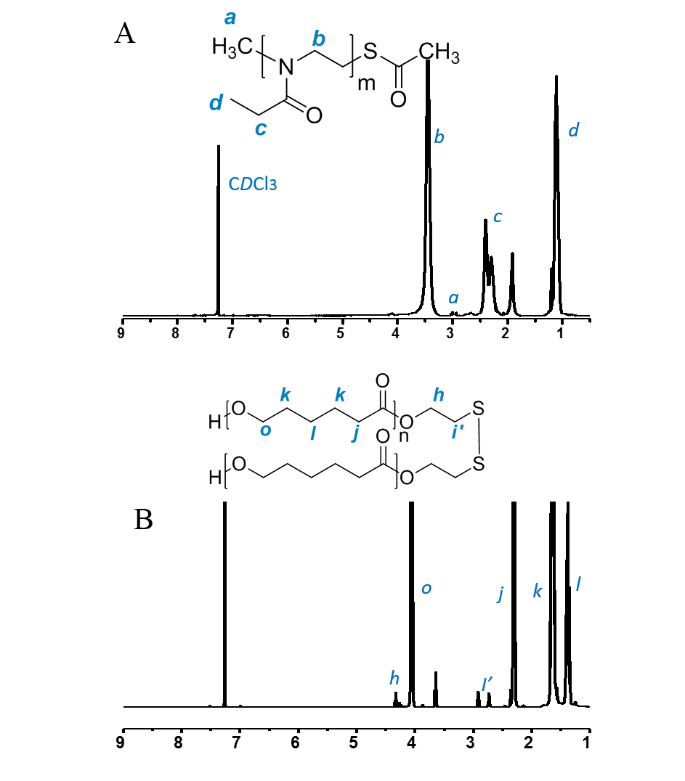


**Figure. S2** ^1^H NMR spectra (400 MHz, C*D*Cl_3_) of PEtOz-SAc (A) and PCL43-SS-PCL43 (B).


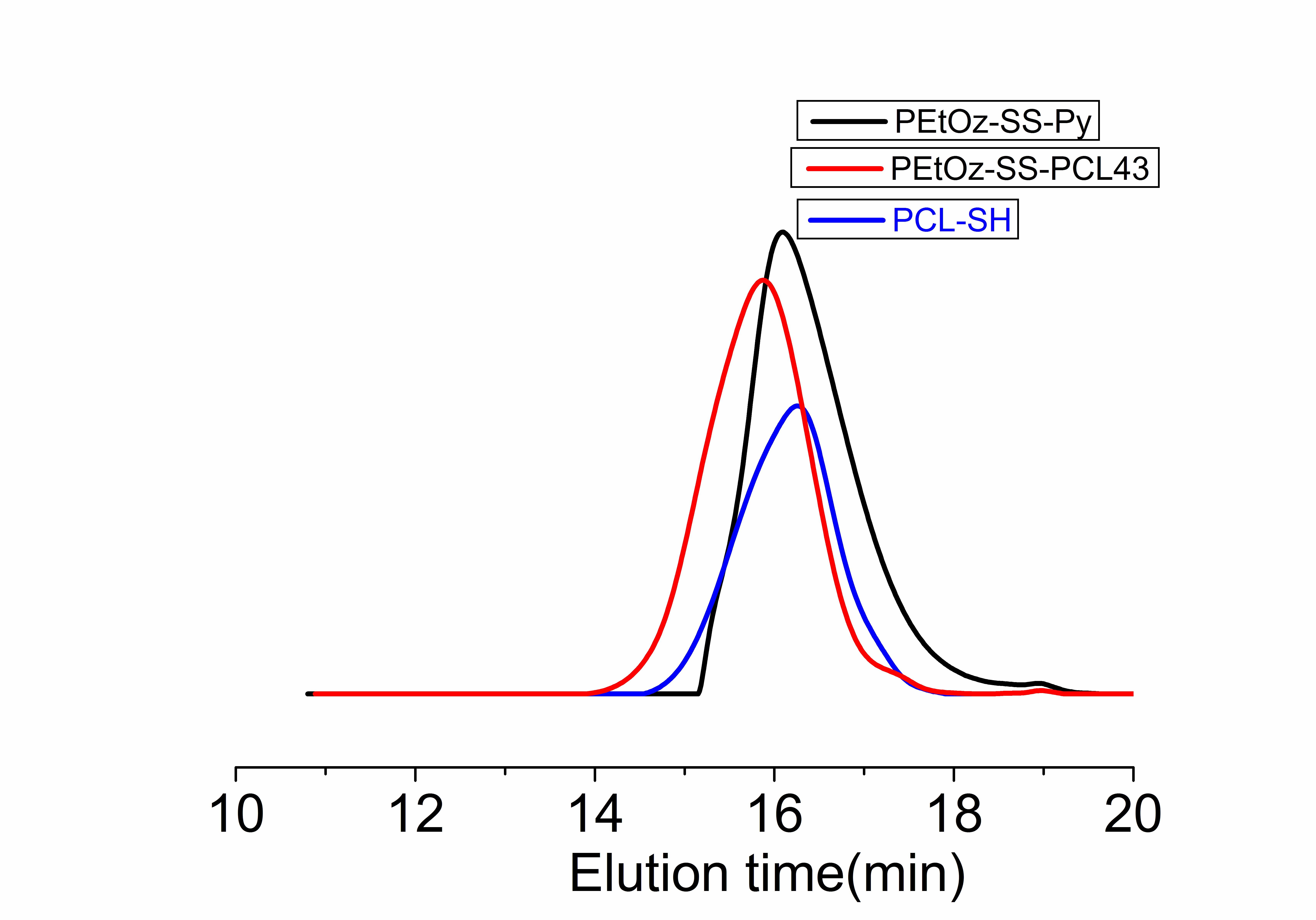


**Figure. S3** GPC profiles of PEtOz-SS-PCL43, PEtOz-SS-Py and PCL-SH.


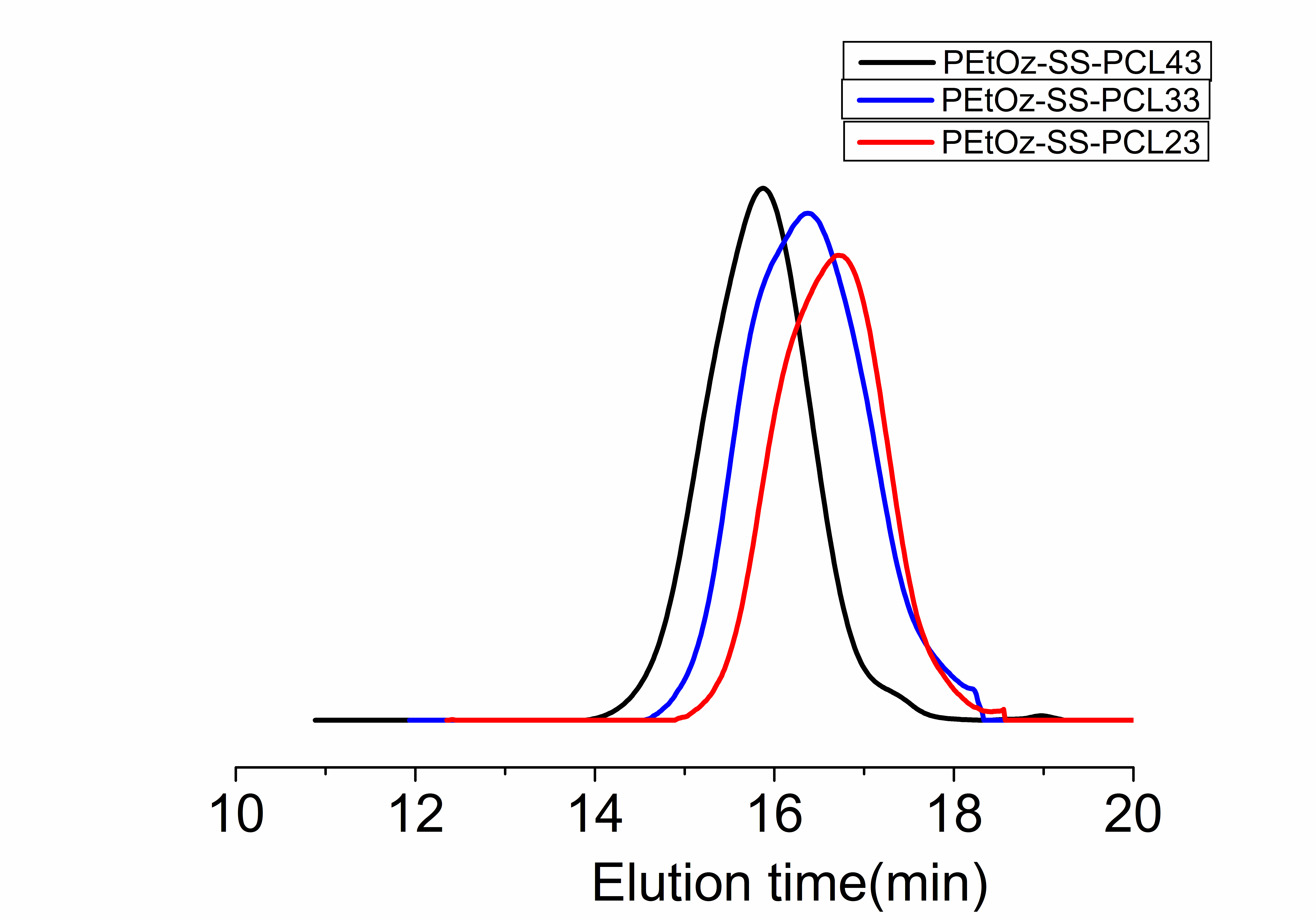


**Figure. S4** GPC profiles of PEtOz-SS-PCL43, PEtOz-SS-PCL33 and PEtOz-SS-PCL23.


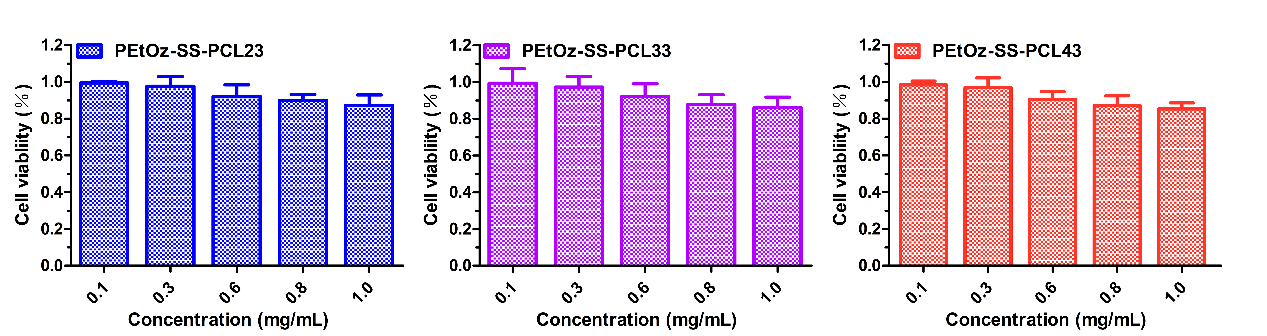


**Figure S5.** Quantitative evaluation of cell survival for C6 cells treated with free PEtOz-SS-PCL23, PEtOz-SS-PCL33 and PEtOz-SS-PCL43 for 24 h by MTT.


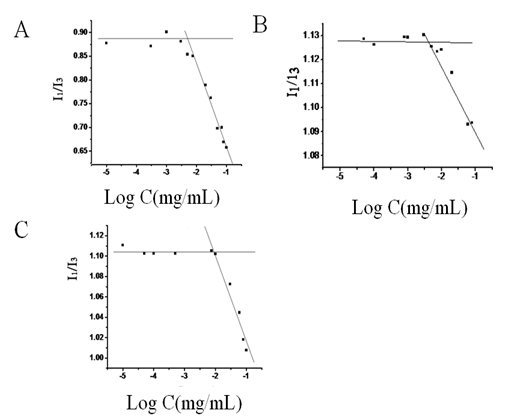


**Figure. S6** The fluorescence intensity ratio I_1_/I_3_ of pyrene as a function of PEtOz-SS-PCL concentration (A: PEtOz-SS-PCL43, B: PEtOz-SS-PCL33, C: PEtOz-SS-PCL23).

**Table S1.** Characterization of blank and DOX loaded PEtOz-SS-PCL micelles (theoretical DLC = 20 wt %)

| Sample | Blank micelles | | |  | DOX-loaded micelles | | DLC  (wt %)*^b^* | DLE  (%)*^b^* | CMC  (mg/L)*^c^* |
| --- | --- | --- | --- | --- | --- | --- | --- | --- | --- |
|  | Size (nm)*^a^* | PDI*^a^* | Zeta (mv)*^a^* |  | Size(nm)*^a^* | PDI*^a^* |  |  |  |
| PEtOz-SS-PCL43 | 97.3±1.8 | 0.23 | -37.8±1.0 |  | 88.4±2.7 | 0.20 | 11.9 | 59.4 | 5.18 |
| PEtOz-SS-PCL33 | 140.6±1.6 | 0.18 | -28.0±0.8 |  | 137.8±1.3 | 0.18 | 10.9 | 54.6 | 6.62 |
| PEtOz-SS-PCL23 | 160.2±1.4 | 0.26 | -19.8±1.4 |  | 162.4±0.1 | 0.12 | 10.1 | 50.3 | 8.29 |

*^a^* Determined by DLS at 25 °C.

*^b^* Determined by fluorescence measurements.

*^c^* Determined using pyrene as a hydrophobic fluorescent probe.

***
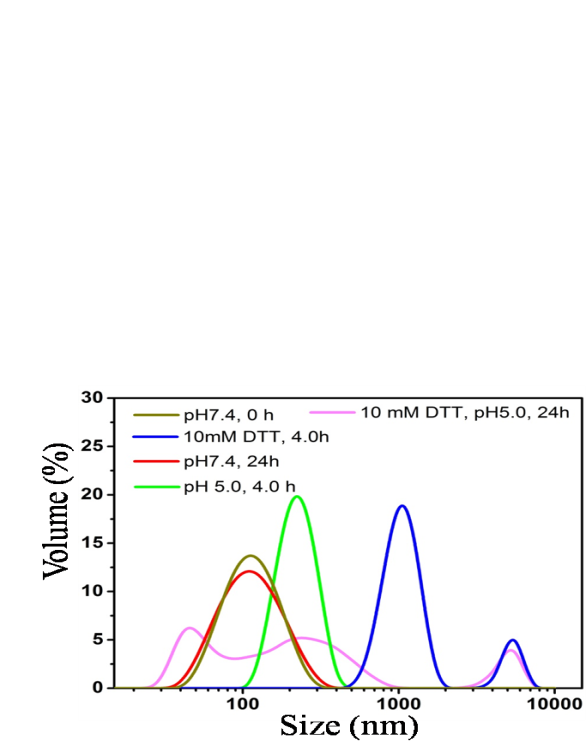
***

**Figure. S7** The size change of PEtOz-SS-PCL43 micelles in response to reduction and pH determined by DLS measurements.


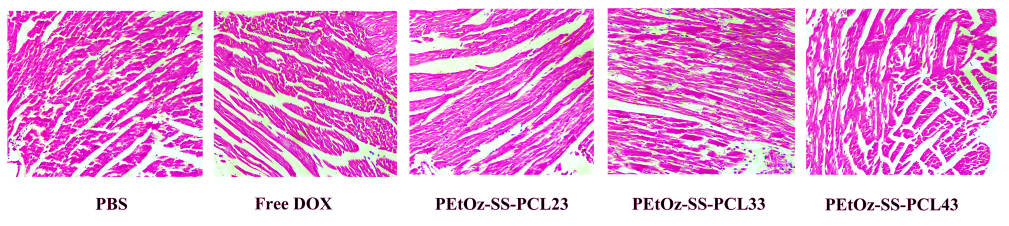


**Figure. S8** Histopathology of H&E-stained myocardial tissue from C6-bearing ICR mice treated with different formulations. All images were analyzed by microscopy at 400 magnification.

**References**

Hsiue GH, Wang CH, Lo CL, et al. (2006). Environmental-sensitive micelles based on poly(2-ethyl-2-oxazoline)-b-poly(L-lactide) diblock copolymer for application in drug delivery. Int J Pharm 317:69-75.

# Sun H, Guo B, Cheng R, et al. (2009). Biodegradable micelles with sheddable poly (ethylene glycol) shells for triggered intracellular release of doxorubicin. Biomaterials 30:6358-6366.
